# Epstein Barr Virus Interleukin 10 Suppresses Anti-inflammatory Phenotype in Human Monocytes

**DOI:** 10.3389/fimmu.2018.02198

**Published:** 2018-10-09

**Authors:** Neelakshi R. Jog, Eliza F. Chakravarty, Joel M. Guthridge, Judith A. James

**Affiliations:** ^1^Arthritis and Clinical Immunology, Oklahoma Medical Research Foundation, Oklahoma City, OK, United States; ^2^Departments of Medicine and Pathology, University of Oklahoma Health Science Center, Oklahoma City, OK, United States

**Keywords:** Epstein Barr virus, monocytes, IL-10, inflammation, autoimmunity, systemic lupus erythematosus

## Abstract

Epstein Barr virus (EBV) is a gamma herpes virus associated with certain malignancies and autoimmune diseases. EBV maintains latency in B cells with occasional reactivation, in part by overcoming the host immune response with viral homologs of several human proteins. EBV interleukin 10 (vIL-10), a lytic phase protein, is a homolog of human IL-10 (hIL-10). The effect of vIL-10 on human monocytes, which are one of the first immune cells to respond to infection, is not known. To understand the role of vIL-10, monocytes from peripheral blood mononuclear cells were stimulated with hIL-10 or vIL-10. Human IL-10 stimulated STAT3 phosphorylation, which is required for suppression of inflammatory responses. However, vIL-10 induced significantly lower phosphorylation of STAT3 compared to hIL-10, and was less efficient in downregulating inflammatory genes. vIL-10 significantly reduced the expression of scavenger receptor CD163 on monocytes, suggesting inhibition of M2 polarization. Furthermore, uptake of apoptotic cells was reduced in vIL-10-stimulated monocytes compared to hIL-10-stimulated monocytes. A neutralizing antibody to IL-10R1 inhibited STAT3 phosphorylation induced by either hIL-10 or vIL-10, suggesting that vIL-10 signals through IL-10R1. Interestingly, vIL-10 suppressed hIL-10-induced STAT3 phosphorylation and inhibited upregulation of suppressors of inflammatory response by hIL-10. We further show that vIL-10 levels were significantly higher in plasma samples from systemic lupus erythematosus (SLE) patients compared to matched unaffected controls. vIL-10 levels did not correlate with hIL-10 levels, but were associated with levels of IgA antibodies to EBV viral capsid antigen, which is an indirect measure of viral reactivation. We propose that the suppression of hIL-10- induced anti-inflammatory genes by vIL-10, together with an increase in inflammatory gene expression, may overcome the anti-inflammatory effects of hIL-10 and exacerbate autoimmune responses in systemic autoimmune diseases.

## Introduction

Epstein Barr virus (EBV) is a highly prevalent gamma herpes virus with over 90% of the adult population being previously exposed to the virus. EBV has been associated with a broad spectrum of carcinomas and lymphoproliferative states, including nasopharyngeal carcinomas, Burkitt's lymphoma, and Hodgkin's lymphoma, as well as with autoimmune diseases such as systemic lupus erythematosus and multiple sclerosis (1–5). EBV maintains latency in B cells and demonstrates occasional reactivation, which can be measured indirectly by serology with increased levels of IgG and IgA responses toward EBV viral capsid antigen (VCA) and EBV early antigen (EA) (6).

EBV encodes homologs of cellular cytokines that allow the virus to escape or curtail host anti-viral responses and to establish latency. One such protein is viral interleukin 10 (vIL-10), a homolog of human interleukin 10 (hIL-10). vIL-10, a late lytic phase protein encoded by the BCRF-1 gene, shares ~80% homology with hIL-10 (7–9). hIL-10 is the founding member of the class II cytokine family, which also includes IL-19, IL-20, IL-22, IL-24, and IL-26 (10). IL-10 is a unique class II cytokine because it potently inhibits the production of pro-inflammatory cytokines such as IFNγ, TNFα, IL-1β, and IL-6, prevents dendritic cell (DC) maturation and inhibits the expression of MHC and co-stimulatory molecules on myeloid cells. hIL-10 is a potent growth and differentiation factor for B-cells, mast cells and thymocytes.

vIL-10 shares some of the suppressive and stimulatory functions of hIL-10. vIL-10 can inhibit inflammatory cytokine (i.e., IFNγ) production and can promote proliferation and differentiation of B cells, as well as immunoglobulin production (7, 8, 11). Functional differences between hIL-10 and vIL-10 have also been reported. vIL-10 cannot co-stimulate mouse thymocyte proliferation or mast cell proliferation and cannot up-regulate MHC class II on B cells (11–13). Innate immunity is the first line of defense against infections; however, the role of vIL-10 in monocyte function has not been determined.

Systemic lupus erythematosus (SLE) is a systemic autoimmune disease characterized by autoantibody generation and immune dysfunction (14). In addition to roles for the adaptive immune system, innate immune cells, such as monocytes, also contribute to disease pathogenesis (15). SLE patients have higher frequencies of EBV infected cells, higher viral loads in blood mononuclear cells, and higher levels of EA IgG (16–18), suggesting more frequent viral reactivation.

In this study we examined whether vIL-10 has similar inhibitory effects on monocytes as hIL-10 and whether vIL-10 is associated with SLE.

## Materials and methods

### Patients

Buffy coats from healthy donors were obtained from the Oklahoma Blood Institute. Coded plasma samples from 20 female SLE patients and 19 matched unaffected controls were obtained from the Oklahoma Rheumatic Disease Research Cores Center. Patients and controls consented to rheumatic diseases research at the time of blood draw. The SLE disease activity index (SLEDAI) (19) ranged from 0-18 (median ± SD, 6 ± 5.65, Table 1, Supplementary Table 1). All subjects gave written informed consent in accordance with the Declaration of Helsinki. The protocol was approved by the Institutional Review Board of the Oklahoma Medical Research Foundation.

**Table 1:** Study participants.

	**SLE patients (*n* = 20)**	**Controls (*n* = 19)**
Age, Mean ±SD	38.25 ± 11.62	37.05 ± 12.27
Sex, n (%)	20 (100)	19 (100)
Race, n (%)		
European American	10 (50)	10 (52.63)
African American	10 (50)	9 (47.7)
SLEDAI, Median ± SD (Range)	6 ± 5.65 (0–18)	N.A.^a^
Complement C3 levels, Median mg/dL, (SD)	115 (44.46)	N.D.^b^
Complement C4 levels, Median mg/dL, (SD)	23 (14.38)	
CH50	26 (18.94)	
**Autoantibody positivity, n (%)**		N.D.
Anti-dsDNA	8 (40)	
Anti-Ro/SSA	5 (25)	
Anti-La/SSB	1 (5)	
Anti-Sm	1 (5)	
Anti-nRNP	5 (25)	
aCL	9 (45)	
**ACR**^c^ **organ manifestations, n (%)**		N.D.
Renal	6 (30)	
Arthritis	19 (95)	
Mucocutaneous^d^	18 (90)	
Hematologic	16 (80)	
Serositis	9 (45)	
**Medications, n (% usage)**		N.D.
Corticosteroids^e^	10 (50)	
Hydroxychloroquine	13 (65)	
Immunosuppressants^f^	9 (45)	

a *N.A.: not applicable*.

b *N.D.: not determined*.

c *American College of Rheumatology*.

d *Malar rash, discoid rash, photosensitivity, oral ulcers*.

e *Prednisone, methylprednisolone, depomedrol*.

f *Azathioprine, mycophenolate mofetil, cyclophosphamide*.

### Monocyte cultures and stimulation

Peripheral blood mononuclear cells (PBMCs) were isolated from buffy coats obtained from healthy donors (Oklahoma Blood Institute). Monocytes were enriched from PBMCs using magnetic bead separation (Miltenyi Biotech). Purity was >90% by flow cytometry and viability was >99% by trypan blue exclusion post enrichment. Monocytes were stimulated with 10 ng/ml recombinant hIL-10 (Peprotech) or recombinant vIL-10 (R&D systems) for indicated times. STAT3 phosphorylation was detected by flow cytometry using antibodies directed against phospho-STAT3 Y705 (BD Biosciences). To inhibit signaling through IL-10R, monocytes were stimulated with hIL-10 or vIL-10 in the presence or absence of a neutralizing antibody to IL-10R1 (Clone: 37607, R&D systems), and STAT phosphorylation was measured as above. To differentiate monocytes into macrophages, cells were cultured with 50 ng/ml M-CSF (R&D systems). On day 6 cells were additionally stimulated with IFNγ (20 ng/ml, Peprotech), IL4 (20 ng/ml, Peprotech), hIL-10 (10 ng/ml), or vIL-10 (10 ng/ml) for 24 h. Surface markers were stained using following antibodies: CD14 Clone M5E2, CD163 Clone GHI/61, CD32 Clone FLI8.26 (BD Biosciences), CD16 Clone 3G8, CD64 Clone 10.1, HLA-DR Clone LN3, CD86 Clone IT2.2 (BioLegend). Cells were acquired on BD LSR II or BD Celesta (BD Biosciences) and data were analyzed using FlowJo (TreeStar, v10).

### Western blot analysis

Whole cell lysates were prepared from monocytes stimulated with hIL-10 or vIL-10 as above. Protein concentration was determined by Bicinchoninic Acid protein assay (Pierce BCA protein assay, Thermo Fisher Scientific). Thirty micrograms of proteins were loaded on a 10% polyacrylamide gel and the separated proteins were transferred to nitrocellulose membrane. pSTAT3 Y705 was detected using rabbit anti pSTAT3 (Y705) polyclonal antibody (Cell Signaling Technologies) and horseradish peroxide conjugated goat anti-rabbit secondary antibody (Jackson ImmunoResearch Laboratories). Total STAT3 was visualized using mouse monoclonal antibody directed toward total STAT3 (Clone:124H6, Cell Signaling Technologies) and horseradish peroxide conjugated goat anti-mouse secondary antibody (Jackson ImmunoResearch Laboratories). Band intensities for pSTAT3 and total STAT3 were determined using “Gels” analysis tool of ImageJ as per the standard instructions (https://imagej.nih.gov/ij/docs/menus/analyze.html#gels). STAT3 phosphorylation was determined as ratio of pSTAT3 to total STAT3.

### Gene expression analysis

Monocytes were stimulated with hIL-10 or vIL-10 for 2 h or 6 h. Total RNA was extracted using Qiagen RNeasy kit. RNA integrity was tested on Agilent Bioanalyzer 2100 using Agilent RNA 6000 Pico kit. RNA was reverse transcribed to cDNA using Fluidigm® Reverse Transcription Master Mix and pre-amplified using Fluidigm Preamp master mix. Gene expression of genes involved in cell survival and cell death, inflammatory and anti-inflammatory immune responses, including transcription factors, cytokines, chemokines and receptors was determined using Delta Gene Assays on Biomark HD (Fluidigm). *GAPDH* was used as an internal/housekeeping control to normalize Ct values. Genes with detectable signal in less than 95% of samples were excluded. Data are represented as 2^−ddCt^ when compared to unstimulated cells. Primers for genes tested are listed in Supplementary Table 2.

### Apoptotic cell uptake assay

Monocytes were stimulated with hIL-10 or vIL-10. Jurkat cells were labeled with tetramethylrhodamine (TAMRA, Invitrogen technologies/Thermo Fisher Scientific) and apoptosis was induced using UV irradiation (10 mJ/mm^2^). Apoptosis was confirmed by flow cytometry using Annexin V. This method yielded at least 50% apoptotic cells. Apoptotic Jurkat cells were added to stimulated or unstimulated monocytes at 4:1 (Jurkat:monocyte) ratio and incubated at 37°C for 120 min. Monocytes were washed with PBS to remove free apoptotic cells and were stained for CD14, CD3 (clone UCHT1, BioLegend), CD16, and CD163. Phagocytic monocytes were defined as CD14+CD3-TAMRA+ cells. Samples were acquired using BD Celesta (BD Biosciences) and analyzed using FlowJo (TreeStar, V10).

### Determination of vIL-10 levels

Plasma samples (450 μl) were concentrated using Amicon-Ultra centrifugation filters-3,000 daltons (Millipore), as per the manufacturer's instructions, following removal of high molecular weight proteins by a 100 kD filter. Proteins from equal volumes of concentrated plasma were separated on a 12% SDS polyacrylamide gel and transferred on to a nitrocellulose membrane. The membranes were blocked with 5% non-fat milk for 1 h at room temperature and incubated with mouse monoclonal antibody directed against vIL-10 (R&D systems) overnight at 4°C. Levels of vIL-10 were detected by a horseradish peroxide conjugated goat anti-mouse secondary antibody (Jackson ImmunoResearch Laboratories). The tiff images of western blots were converted to 8-bit image in ImageJ. Bands were quantified using “Gels” analysis tool of ImageJ (https://imagej.nih.gov/ij/docs/menus/analyze.html#gels). Equal volume of concentrated pooled sera from infectious mononucleosis patients (Discovery Life Sciences) was loaded on each gel as a standard to account for gel-to-gel variation. The band intensity for vIL-10 in SLE or control samples was normalized to the standard on each gel. Rabbit anti-human IL-10 monoclonal antibody (D13A11 Cell Signaling Technologies), horseradish peroxide conjugated goat anti-rabbit secondary antibody (Jackson ImmunoResearch Laboratories), and recombinant human IL-10 (Peprotech) were used to test the specificity of the vIL-10 antibody. hIL-10 levels were measured by xMAP Luminex assay (Procarta xMAP Platinum IL-10, Affymetrix).

### Autoantibody testing

Autoantibody testing was performed by the CAP-certified/CLIA-approved OMRF Clinical Immunology Laboratory as described previously (20, 21). Anti-nuclear antibodies (ANA) and anti-double stranded DNA (dsDNA) antibodies were determined by indirect immunofluorescence using Hep2 cells or *Crithidia luciliae*, respectively (INOVA Diagnostics, San Diego, CA). Positivity was defined as ANA detection at a titer of ≥ 1:120 and anti-dsDNA detection at ≥ 1:30. Antibodies against extractable nuclear antigens (Ro, La, Sm, nRNP, and ribosomal P) were detected by immunodiffusion. Anti-cardiolipin (aCL) antibodies were measured by enzyme-linked immunosorbent assay (ELISA). Positivity was defined as >20 IgG units.

### Anti-viral antibody testing

Antibodies against Epstein Barr virus viral capsid antigen (VCA, IgG), Epstein Barr virus early antigen (EA, IgG), (Alere-Wampole), and VCA IgA (Calbiotech) were measured using commercial ELISAs according to manufacturer's instructions. Optical density was measured using EMax® Plus Microplate reader (Molecular Devices). Standard calibrators used in each assay were used to calculate index values/optical density (OD) ratios, which serve as semi-quantitative measure of antibody levels. All assays met pre-determined quality control measures based upon positive, negative, and blank controls.

### Statistical analyses

Differences between groups were determined by Wilcoxon test or Mann-Whitney test as appropriate. ANOVA was used when more than two groups were compared, and differences between groups were determined by Tukey *post hoc* test. Sample size (*n*) for all experiments represents number of independent donors. Each experiment was performed at least twice with different numbers of donors in each experiment to confirm reproducibility. Statistical significance was defined as *p* < 0.05. All analyses were performed using Prism 7 version 7.0b (Graphpad Software, Inc).

## Results

### Compared to hIL-10, vIL-10 stimulates lower levels of STAT3 phosphorylation in monocytes

**Figure 1:**
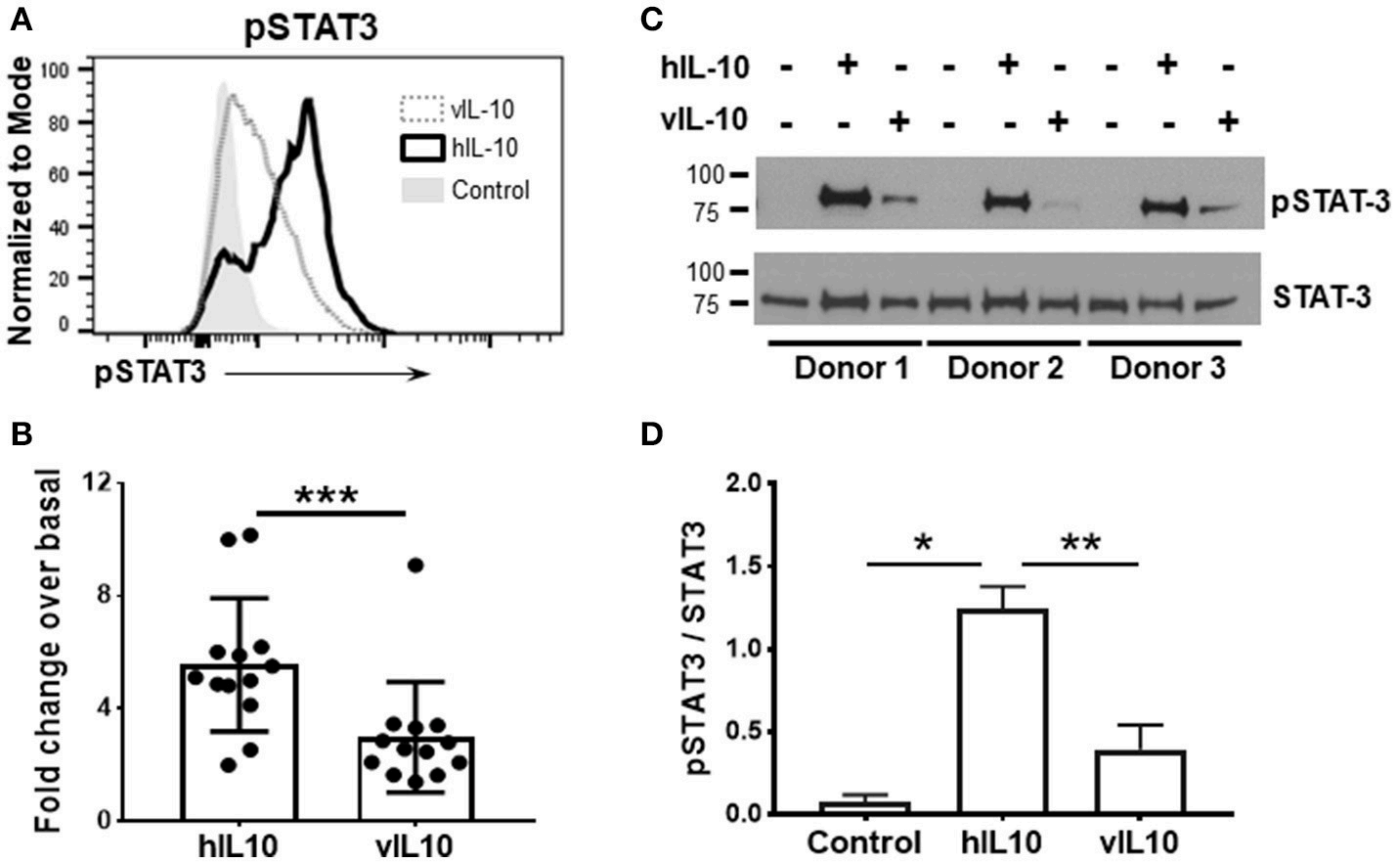
vIL-10 induces limited STAT3 phosphorylation in monocytes. Monocytes were enriched from PBMCs using magnetic beads and stimulated with 10 ng/ml hIL-10 or vIL-10 for 30 min. pSTAT3 (pY705) was measured by flow cytometry. **(A)** Representative histogram for pSTAT3. **(B)** Mean fluorescence intensities for pSTAT3 are shown as fold increase over unstimulated. Data are presented as mean ± SD, *n* = 13, ^***^p < 0.001 by Wilcoxon test. **(C)** Western blot analysis shows increased STAT3 phosphorylation in cells stimulated with hIL-10 compared to those stimulated with vIL-10. Cells from three independent donors were used. Upper panel: phosphorylated-STAT3, lower panel: total STAT3. **(D)** pSTAT3 presented as ratio of band intensity of pSTAT3 and STAT3 from **(C)** for each donor. ^*^p < 0.05, ^**^p < 0.01 by ANOVA.

To determine whether vIL-10 has inhibitory effects on monocytes similar to hIL-10, we examined downstream STAT phosphorylation in human monocytes stimulated *in vitro* with human or viral IL-10. IL-10 signals by activating IL-10 receptor-associated Janus kinase 1, and tyrosine kinase 2 protein tyrosine kinases, and downstream STATs. The inhibitory effects of IL-10 in myeloid cells are predominantly mediated by STAT3, and STAT3 is required for the downregulation of inflammatory genes, and up-regulation of anti-inflammatory genes by IL-10 (22). Therefore, monocytes enriched from healthy PBMCs were stimulated with human or viral IL-10 and phosphorylated STAT3 (pSTAT3) was determined by flow cytometry. Figures 1A,B show that vIL-10 induced significantly lower pSTAT3 compared to hIL-10. We confirmed our data using western blot analyses of whole cell lysates from monocytes stimulated with hIL-10 or vIL-10 for pSTAT3 and total STAT3. Figures 1C,D show that hIL-10 stimulated significantly higher phosphorylation of STAT3 and a higher ratio of pSTAT3 to total STAT3 (Figure 1D). These data suggest differences in regulation of gene expression by vIL10.

### vIL-10 is inefficient in down-regulating pro-inflammatory gene expression

**Figure 2:**
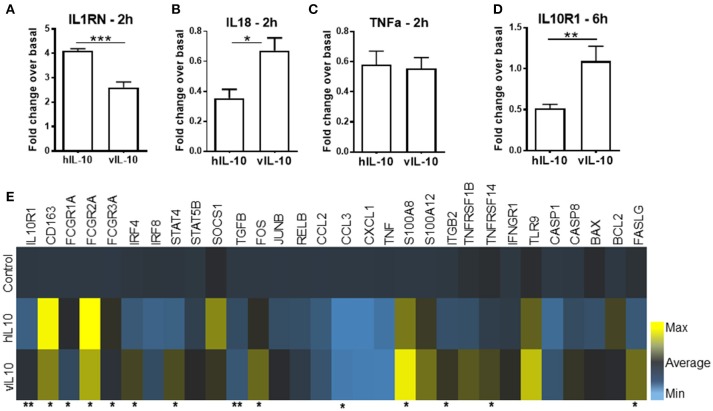
vIL-10 induces a distinct pattern of gene expression in monocytes. Monocytes were stimulated with 10 ng/ml hIL-10 or vIL-10 for 2 h **(A–C)** or 6 h **(D,E)**. Gene expression was determined by quantitative PCR (Biomark HD, Fluidigm). vIL-10 showed **(A)** lower induction of IL1 receptor antagonist gene *IL1RN* and **(B)** lower downregulation of *IL18*. **(C)** Both hIL-10 and vIL-10 downregulated *TNFA*. **(D)** hIL-10 downregulated IL-10 receptor 1 *(IL-10R1*), whereas vIL-10 did not. **(E)** vIL-10 induced higher gene expression of S100A8 proteins, transcription factors, and receptors implicated in inflammatory response. Data are presented as fold change over basal unstimulated cells, such that values >1 indicate an increase and values < 1 indicate a decrease in expression. *n* = 4–8, ^*^p < 0.05, ^**^p < 0.01, ^***^p < 0.001 when comparing level of expression between hIL-10 and vIL-10 stimulated monocytes.

Activation of STAT3 regulates the gene expression induced by IL-10. Since reduced phosphorylation of STAT3 was observed after vIL-10 stimulation, we determined the gene expression profiles induced by hIL-10 or vIL-10 in monocytes. As expected, hIL-10 induced prominent suppression of inflammatory genes (Figure 2). Stimulation with hIL-10 increased expression of the anti-inflammatory interleukin 1 receptor antagonist gene (*IL1RN*), while vIL-10 stimulated a smaller increase in *IL1RN* gene expression (Figure 2A). *IL18* gene expression was downregulated by hIL-10, while vIL-10 was less efficient (Figure 2B). Both hIL-10 and vIL-10 downregulated *TNFA* expression (Figure 2C).

We saw an increase in CD163 gene expression with hIL-10, whereas vIL-10 induced significantly lower upregulation of CD163 (Figure 2E). Suppressor of cytokine secretion-1 (*SOCS1*), a key mediator of IL-10-regulated anti-inflammatory gene expression, was induced by hIL-10, but not by vIL-10 (Figure 2E). vIL-10 did induce higher gene expression of *S100A8, FASLG*, transcription factors such as *IRF4*, and receptors implicated in inflammatory responses (Figure 2E). hIL-10 down-regulated IL-10 receptor 1 (*IL10R1*), whereas vIL-10 did not (Figure 2D). These data support the hypothesis that vIL-10 induces inflammatory gene expression in monocytes and suggest differences are present in signaling pathways regulated by hIL-10 and vIL-10.

### vIL-10 induces a weaker anti-inflammatory monocyte phenotype than hIL-10

**Figure 3:**
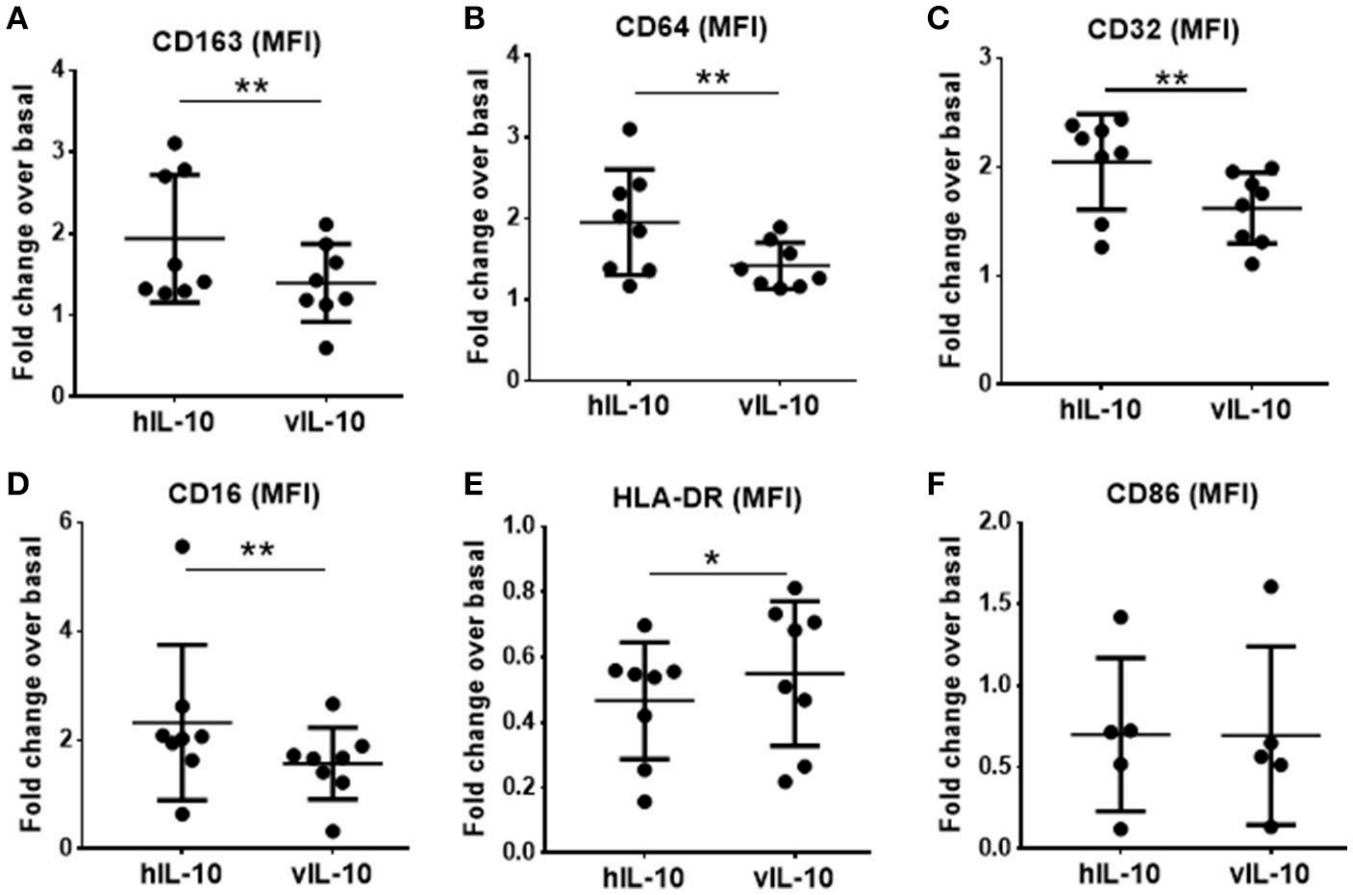
Surface marker expression on monocytes stimulated with hIL10 or vIL10. Monocytes were stimulated with 10 ng/ml hIL-10 or vIL-10 for 18 h, and surface marker expression was determined by flow cytometry with antibodies for **(A)** CD163, **(B)** CD64, **(C)** CD32, **(D)** CD16, **(E)** HLA-DR, and **(F)** CD86. Monocytes stimulated with vIL-10 had smaller increases in CD163, CD64, CD32, and CD16 surface expression **(A–D)** and smaller decreases in HLA-DR surface expression (**E**) when compared to hIL-10. No differences in CD86 were observed between monocytes stimulated with hIL-10 or vIL-10. (**F)** Data are presented as fold change in fluorescence intensity of the marker compared to basal unstimulated cells, such that values >1 indicate an increase and values < 1 indicate a decrease in expression. Each dot represents an independent donor. *n* = 5–8, ^*^p < 0.05, ^**^p < 0.01.

Human IL-10 enhances expression of CD163 and Fc gamma receptor 1 (CD64) and down-regulates class II MHC expression, thereby reducing the antigen presentation capability of monocytes (23, 24). As expected, hIL-10 increased levels of CD163, and Fc gamma receptors (FcγR) CD64, CD32, and CD16 expression on monocytes (Figures 3A–D). The numbers of CD14+ cells expressing high levels of CD163, CD64. CD32 and CD16 were also higher (Supplementary Figure 1). As shown previously, hIL-10 down-regulated HLA-DR expression (Figure 3E). In comparison, monocytes stimulated with vIL-10 showed weaker induction of CD163, CD64, CD32, and CD16, and fewer cells expressed high levels of these markers (Figures 3A–D, Supplementary Figure 1). Interestingly, vIL-10 was less efficient in down-regulating HLA-DR, when compared to hIL-10 (Figure 3E). No significant differences were observed between the levels of CD86 on monocytes stimulated with hIL-10 or vIL-10 (Figure 3F).

**Figure 4:**
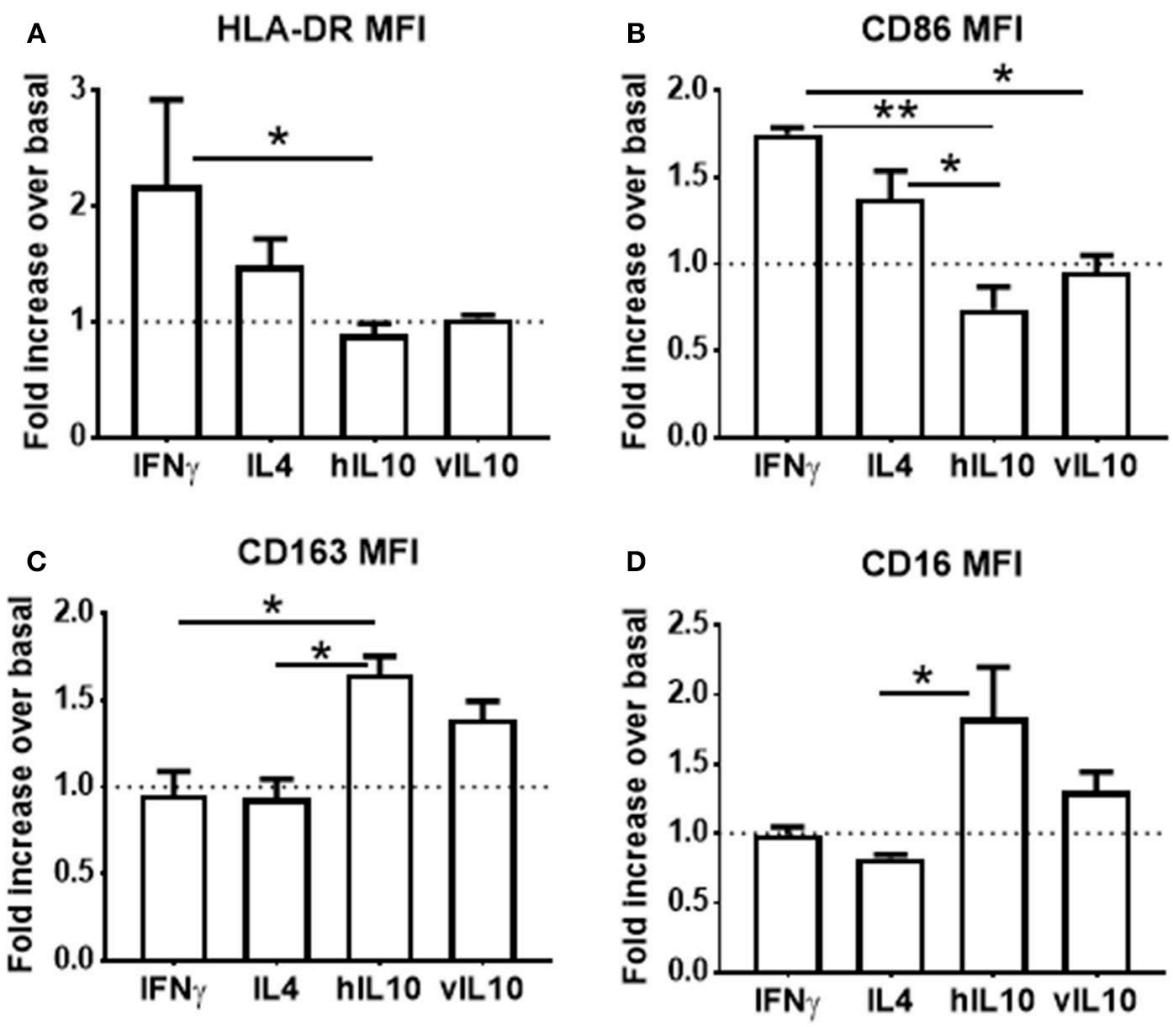
vIL-10 does not redirect macrophage polarization. Monocytes from healthy donors were differentiated into macrophages with M-CSF for 6 days. Macrophages were treated with IFNγ (M1 polarization), IL4 (M2a polarization), hIL-10 (M2c polarization), or vIL-10 for 24 h. Surface expression of **(A)** HLA-DR, **(B)** CD86, **(C)** CD163, and **(D)** CD16 was measured by flow cytometry. Data are presented as fold change over basal, such that values >1 indicate an increase and values < 1 indicate a decrease in expression. *n* = 3, ^*^p < 0.05, ^**^p < 0.01.

Monocyte derived macrophages can be polarized into inflammatory and anti-inflammatory phenotypes (25). Interferon gamma (IFNγ) polarizes macrophages into the pro-inflammatory M1 (classically activated) phenotype, which is characterized by increased expression of co-stimulatory molecules. IL-4 polarizes into the anti-inflammatory M2a phenotype, and IL-10 polarizes into the anti-inflammatory M2c phenotype, which is characterized by reduced expression of co-stimulatory molecules and increased CD163 expression. To determine whether polarization of macrophages induced by hIL-10 and vIL-10 differs, monocytes were differentiated into macrophages using M-CSF. Differentiated macrophages were polarized into M1, M2a, or M2c by stimulating with the appropriate cytokines for 24 h. Figures 4A–D show that we reproduce M1, M2a, and M2c phenotypes with IFNγ, IL4, and hIL-10 stimulation respectively, and the phenotype induced by vIL10 was similar to that induced by hIL-10. These data show that vIL-10 does not skew the polarization of macrophages into the inflammatory M1 phenotype.

### vIL10 stimulated monocytes have reduced ability to uptake apoptotic cells

**Figure 5:**
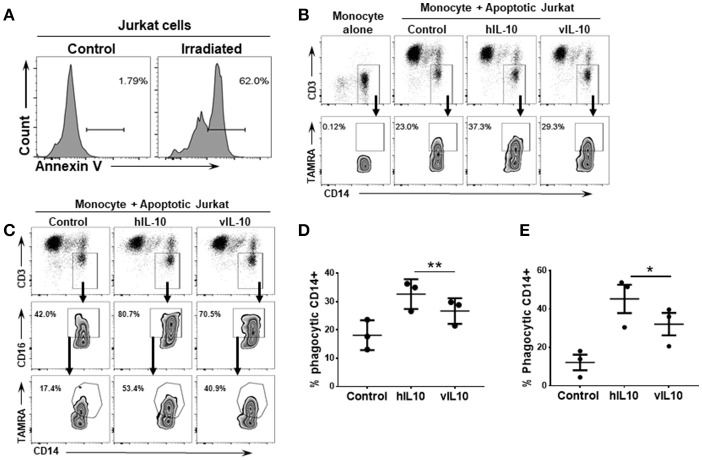
vIL-10 reduces uptake of apoptotic cells by monocytes compared to hIL-10. Apoptosis was induced in Jurkat cells by UV irradiation. **(A)** Representative histogram showing percent of apoptotic cells following UV irradiation. Monocytes were stimulated with hIL-10 or vIL-10 for 18 h, incubated with fluorescently labeled apoptotic Jurkat cells, and assessed for uptake of apoptotic cells by flow cytometry. **(B,C)** Representative gating strategy used to determine number of CD14+ and CD14+CD16+ phagocytic cells. Jurkat cell uptake was lower after vIL-10 stimulation compared to hIL-10 stimulation in **(D)** total monocytes (CD14+) as per gating strategy in **(B)**, and **(E)** CD16hi CD14+ monocytes, as per gating strategy in **(C)**. The data are represented as percent of CD14+CD3- cells. Each dot represents independent donor. *n* = 3, ^*^p < 0.05, ^**^p < 0.01.

Human IL-10 induces an anti-inflammatory phenotype in monocytes with increased phagocytosis (26, 27). To determine whether vIL-10 stimulates similar capacity to uptake apoptotic cells as hIL-10, monocytes treated with hIL-10 or vIL-10 were fed fluorescently labeled apoptotic Jurkat cells (Figure 5A), and internalized fluorescent dye was measured by flow cytometry. Compared to hIL-10-stimulated monocytes, fewer vIL-10-stimulated monocytes internalized apoptotic cells (Figures 5B,D). Decreased uptake can possibly be explained by reduced phagocytosis by CD163 and CD16 expressing cells, as vIL-10 stimulation produced fewer phagocytic CD16^hi^ monocytes compared to hIL-10 stimulation (Figures 5C,E). These data further support a suppressed anti-inflammatory phenotype with vIL-10 stimulation.

### vIL-10 can inhibit effects of hIL-10 on monocytes

**Figure 6:**
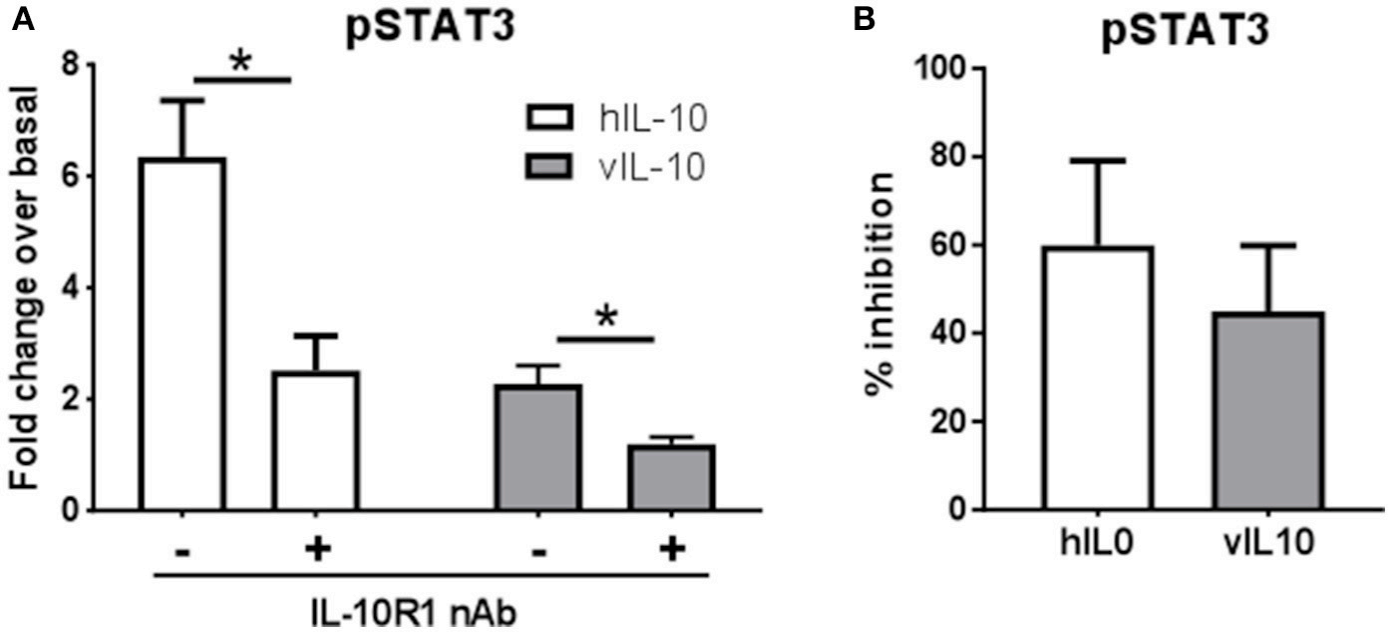
vIL-10 signals through IL-10R1 in human monocytes. Monocytes were stimulated with 10 ng/ml hIL-10 or vIL-10 for 30 min in the presence or absence of 1 μg/ml anti-IL-10R1 antibody. pSTAT3 (pY705) was measured by flow cytometry. **(A)** Data are represented as fold increase over unstimulated, **(B)** Inhibition of pSTAT3 was calculated as the percent reduction of pSTAT3 levels in the presence of neutralizing antibody compared to cells not treated with neutralizing antibody. *n* = 5, ^*^p < 0.05.

**Figure 7:**
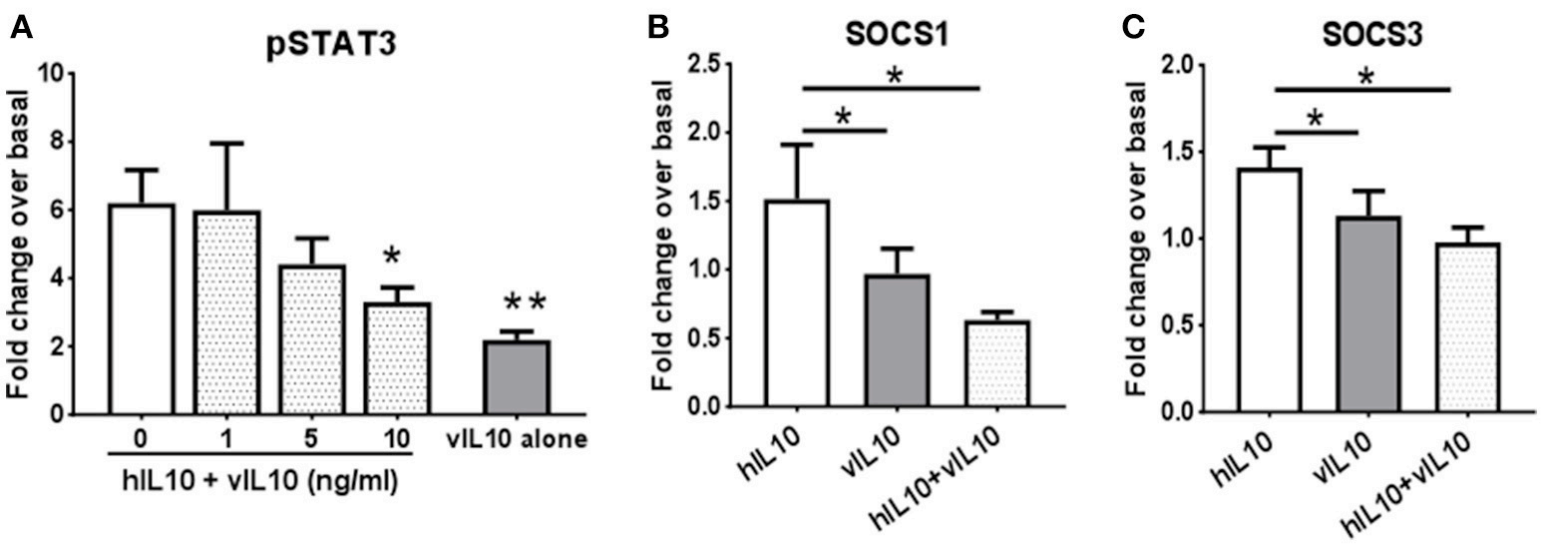
vIL-10 inhibits hIL-10 signaling in monocyte. **(A)** Monocytes were stimulated with either hIL-10 (10 ng/ml) alone, hIL-10 (10 ng/ml) in the presence of increasing concentrations of vIL-10 (1, 5, 10 ng/ml), or vIL-10 alone (10 ng/ml) for 30 min. pSTAT3 was measured by flow cytometry. *n* = 4. **(B,C)** Monocytes were stimulated with 10 ng/ml hIL-10, vIL-10, or hIL-10+vIL-10 for 6 h. Gene expression was determined by quantitative PCR (Biomark HD, Fluidigm). Data show that vIL-10 inhibits the effects of hIL-10 in monocytes. *n* = 4, ^*^p < 0.05, ^**^p < 0.01 when compared to monocytes stimulated with hIL10 alone.

We saw an inability of vIL-10 to downregulate IL-10R1. To understand whether vIL-10 signals through IL-10R1 or uses an alternate receptor, we stimulated monocytes with hIL-10 or vIL-10 in the presence or absence of a neutralizing antibody to IL-10R1. Although vIL-10 induced significantly lower pSTAT3 compared to hIL-10, the IL-10R1 neutralizing antibody was able to reduce STAT3 phosphorylation induced by vIL-10 (Figure 6A). The percent inhibition of STAT3 phosphorylation by neutralizing IL-10R1 antibody was not different between hIL-10 and vIL-10 stimulated monocytes (Figure 6B). These data show that vIL-10 uses IL-10R1 similar to hIL-10. To further understand whether vIL-10 competes with hIL-10 for the receptor, we stimulated monocytes with hIL-10 in the presence of increasing concentrations of vIL-10. Interestingly, vIL-10 reduced hIL-10 induced STAT3 phosphorylation to levels similar to cells stimulated with vIL-10 alone (Figure 7A). Furthermore, vIL-10 inhibited hIL-10-stimulated *SOCS1* and *SOCS3* gene expression (Figures 7B,C). Together, these results suggest that vIL-10 can block hIL-10-induced anti-inflammatory responses in monocytes through competitive inhibition.

### SLE patients have increased levels of plasma vIL-10

SLE patients have aberrant responses to EBV infection and evidence of increased EBV reactivation (16–18). vIL-10 is a late lytic phase protein and is expected to be higher during viral reactivation. However, whether the levels of vIL-10 are higher in SLE patients has not yet been determined. We therefore measured circulating vIL-10 levels in SLE patients and unaffected controls.

**Figure 8:**
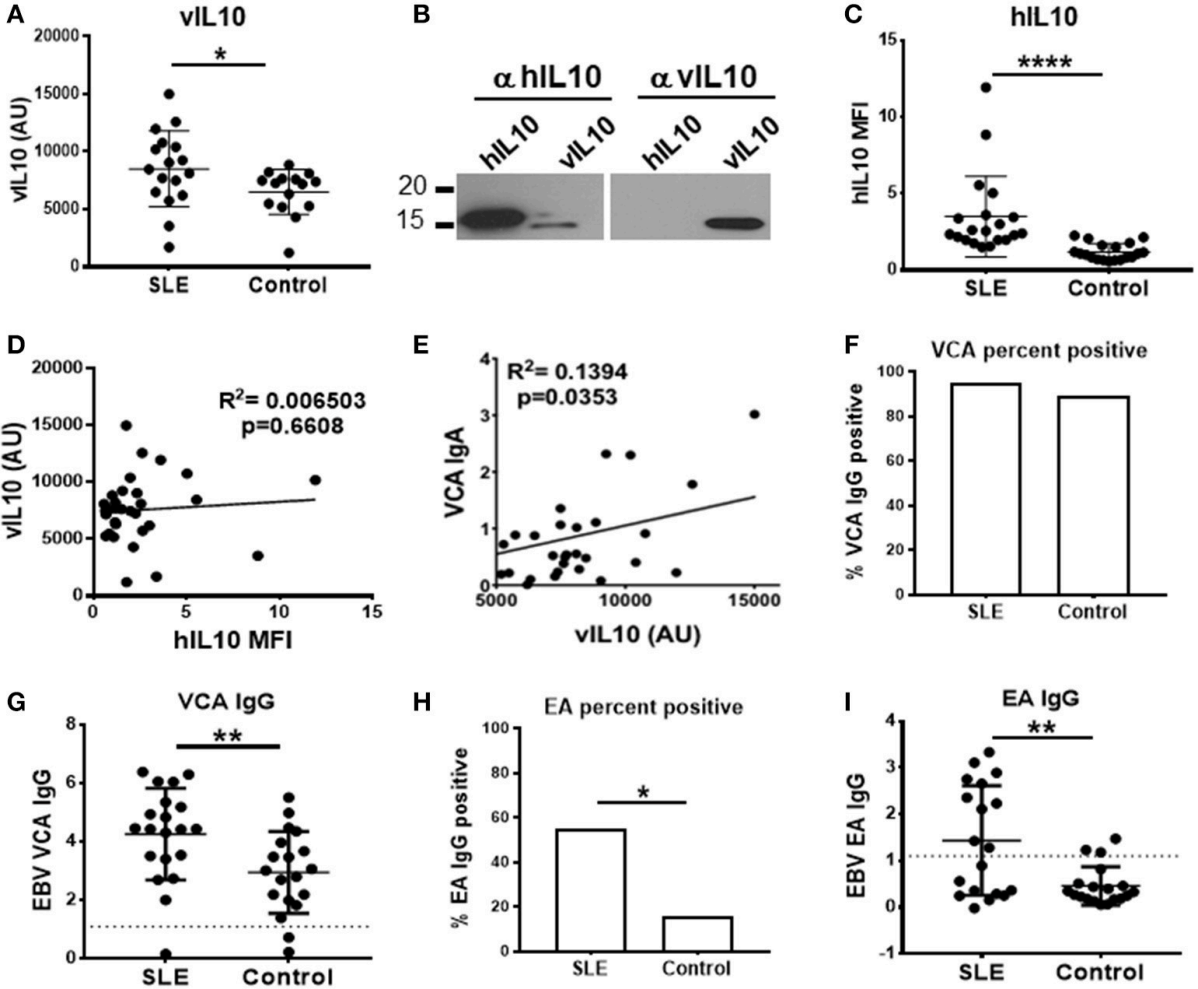
SLE plasma samples show increased levels of vIL-10. **(A)** Plasma from SLE patients and controls were concentrated and vIL-10 measured by Western blot. **(B)** The specificity of anti-vIL-10 antibody was tested by western blots of recombinant hIL-10 and vIL-10. A representative blot is shown. Antibody against hIL-10 detected both human and viral IL-10, however anti-vIL-10 antibody only detected vIL-10 and not hIL-10. **(C)** hIL-10 levels were measured by xMAP Luminex assay (Procarta xMAP Platinum IL-10, Affymetrix). **(D)** No correlation was observed between vIL-10 and hIL-10, measured as in **(A,C)**, respectively, in either SLE or control samples. **(E)** Levels of anti-VCA IgA, measured by ELISA, correlated with vIL10. **(F,G)** Anti-VCA IgG was measured by ELISA as a marker of previous exposure to EBV in SLE and control samples. **(H,I)** SLE patients had higher anti-EA IgG positivity **(H)** and higher levels of anti-EA IgG **(I)** by ELISA, suggesting increased viral reactivation in SLE patients. ^*^p < 0.05, ^**^p < 0.01, ^****^p < 0.0001 by Mann Whitney.

SLE patients had significantly higher levels of vIL-10 compared to unaffected controls (Figure 8A). The antibody used to detect vIL-10 was unable to detect hIL-10 (Figure 8B). SLE patients also showed higher levels of hIL-10 (Figure 8C), but levels of vIL-10 did not directly correlate with hIL-10 levels (Figure 8D). However, vIL-10 levels did associate with IgA responses to viral capsid antigen (VCA), a measure of active infection or viral reactivation (Figure 8E).

Infection with EBV leads to IgG responses to VCA, which persist for a lifetime, albeit at a lower concentration than seen in acute infection or reactivation. To determine whether SLE patients and controls had previous EBV exposure, we measured VCA IgG in plasma. The VCA IgG seropositivity, indicative of prior exposure to EBV, was similar between SLE patients and controls (Figure 8F), although SLE patients did have higher VCA IgG levels (Figure 8G). SLE patients have previously been shown to have higher levels of IgG antibodies to EBV early antigen (EA), which are detectable during active infection or recent reactivation (6, 16, 28). In our cohort, as reported previously, SLE patients showed significantly higher EA IgG seropositivity (Figure 8H), and significantly higher EA IgG levels compared to controls (Figure 8I) (29), indicative of more frequent reactivation of EBV in SLE patients. These data suggest that frequent reactivation of EBV in SLE patients may lead to increased vIL-10 levels in circulation.

## Discussion

Our data show that, unlike hIL-10, vIL-10 is inefficient in up-regulation of anti-inflammatory genes such as *IL-1RN* and *SOCS1*. On the other hand, vIL-10 induces expression of transcription factors such as IRF4, STAT5, AP1, which is not observed with hIL-10. Monocytes stimulated with vIL-10 have reduced CD163 and FcγR expression compared to hIL-10 stimulated cells and are defective in clearance of apoptotic cells. Further, vIL-10 directly inhibits induction of anti-inflammatory gene expression induced by hIL-10. Thus, in contrast with hIL-10, vIL-10 stimulates monocytes to cells that are more pro-inflammatory with reduced ability to phagocytose dying cells.

The mechanisms by which vIL-10 induces differential activation of monocytes is not clear. vIL-10 has ~1,000-fold lower affinity for the IL10R than hIL-10 (7). The differences we saw with some genes (*S100A8, CCL3/MIP1A)* were mostly in the magnitude as would be expected with reduced affinity to the receptor. However, compared to unstimulated cells, vIL-10 upregulated gene expression of several proteins such as IRF4, STAT4, and TNFRSF14, FASLG whereas hIL-10 downregulated these genes. These changes in gene expression suggest differences in downstream signaling cascades, and possibly receptor engagement. Interestingly, the soluble form of IL-10 receptor (sIL-10R) can reverse the inhibitory effects of hIL-10, but not vIL-10, suggesting that vIL-10 may engage different receptor subunits for signaling (7). However, our data with neutralizing antibodies to IL-10R1 suggest that vIL-10 signals through IL-10R1, and furthermore can compete with and inhibit hIL-10 signaling through the receptor.

Our data suggest that a lytic phase protein can alter the functions of innate immune cells, which could potentially contribute to EBV persistence. We show that vIL-10, which is expressed during lytic replication of EBV, reduces the ability of monocytes to clear apoptotic cells. Accumulation of apoptotic cells can lead to increased secondary necrosis. Uptake of necrotic cells by dendritic cells (DCs) results in better antigen processing and presentation, which initiates the adaptive arm and possibly allows the virus to establish latency.

Although vIL-10 induces a relatively inflammatory phenotype in monocytes, it does not polarize macrophages into inflammatory M1 macrophages, based on surface marker expression. However, whether the macrophages polarized by vIL-10 are less efficient anti-inflammatory macrophages compared to hIL-10 or whether vIL-10 polarizes macrophages to a phenotype that has not been characterized yet, is not clear and needs further assessment into the function of these macrophages.

EBV has been consistently associated with SLE, but the precise role of EBV or vIL-10 in SLE pathogenesis is not clear. The frequent EBV reactivation reported in SLE patients and the increase in vIL-10 seen in this small cohort suggest that vIL-10 may have implications in SLE pathogenesis. The ability of vIL-10 to inhibit hIL-10 signaling suggests that increased vIL-10 in SLE patients may be able to overcome the inhibitory effects of hIL-10 on immune responses, thereby exacerbating autoimmune responses. Plasma vIL-10 levels did not correlate with SELDAI scores (Supplementary Figure 2). This may require studies of patients with broader ranges of disease activity and different organ manifestations to form conclusions. Analysis of longitudinal samples from SLE patients may be necessary to evaluate the role of vIL-10 in SLE disease progression.

The deficiency of receptors involved in clearance of dying cells such as TAM receptors, TIM-4, and MFGE8 receptor is associated with the development of lupus like symptoms in mice (30–33). Furthermore, macrophages from SLE patients show impaired uptake of dying cells (34). Although the exact mechanisms are not completely understood, ineffective clearance results in accumulation of cell debris due to rupture of apoptotic cells that may lead to increased inflammation and antigen presentation by dendritic cells (DCs) (35). Our data show that vIL-10 induces genes that are implicated in dendritic cell differentiation (IRF4, STAT4, STAT5) (36). Monocytes cultured with vIL-10 also showed lower CD163 and FcγRI expression and inefficient down-regulation of HLA-DR compared to hIL-10. These data suggest that vIL-10 may differentiate monocytes into cells with increased antigen presentation capability, but reduced clearance ability.

We propose that the inflammatory environment induced by vIL-10 and self-antigen presentation in the context of genetic predisposition to autoimmunity may lead to loss of tolerance and amplification of autoimmune responses in SLE patients.

## Author contributions

All authors contributed to drafting or revising article critically for intellectual content, and approved the final version of the article to be published. NJ and JJ: Substantial contributions to study conception and design; NJ, EC, JG, and JJ: Substantial contributions to acquisition of data; NJ and JJ: Substantial contributions to analysis and interpretation of data.

### Conflict of interest statement

The authors declare that the research was conducted in the absence of any commercial or financial relationships that could be construed as a potential conflict of interest.
