# Supplementary material for: Epstein Barr Virus Interleukin 10 Suppresses Anti-inflammatory Phenotype in Human Monocytes
Source: Front Immunol. 2018 Oct 9;9:2198. doi: 10.3389/fimmu.2018.02198 (PMC6189329; doi:10.3389/fimmu.2018.02198)
Supplement: Supplementary file 1 [file Data_Sheet_1.pdf]

Supplementary Material

Epstein Barr Virus Interleukin 10 Suppresses Anti-inflammatory Phenotype in Human Monocytes

Neelakshi R. Jog, Eliza F. Chakravarty, Joel M. Guthridge, Judith A. James*

***Correspondence:** Judith A. James, M.D., Ph.D. Email: judith-james@omrf.org

Supplementary Table 1. Other clinical manifestations in SLE patient cohort

	**SLE patients (n=20)**
Livedo	3 (15)
Lymphadenopathy	1 (5)
Diabetes	2 (10)
Cancer	1 (5)
Migraines (headaches)	9 (45)
Neurological (seizures, chorea, psychosis, cerebritis)	3 (15)
Raynauds	18 (90)
Myalgia	10 (50)
Fatigue	18 (90)
Alopecia	16 (80)
Cutaneous vasculitis	12 (60)
Peripheral neuropathy (parathesias, etc)	2 (10)
Sicca	12 (60)
Low complement	17 (85)
Arthralgia	15 (75)
Leukopenia	9 (45)
lymphopenia	7 (35)
Neutropenia	1 (5)
Anemia	6 (30)
APS	5 (25)
Angina	1 (5)
Angioedema	1 (5)
Discoid lupus	1 (5)
Thyroid disease	2 (10)
Pancreatitis	1 (5)
Adenopathy	1 (5)
Fever	11 (55)

Supplementary Table 2. Primers used for quantitative PCRs

Gene	Forward Primer	Reverse Primer
SOCS1	CATCCGCGTGCACTTTCA	GCTCGAAGAGGCAGTCGAA
SOCS3	TTCAGCTCCAAGAGCGAGTA	TCACTGCGCTCCAGTAGAA
IL10RA	CGCCGAAAGAAGCTACCC	CGCTGGCTGATGAAGATGAA
IL1RN	CCGACCCTCTGGGAGAAAA	CCTCAGATAGAAGGTCTTCTGGTTA
IL18	ACCAAGGAAATCGGCCTCTA	ACCTCTAGGCTGGCTATCTTTA
IRF4	CACCATGACAACGCCTTACC	CGAGGGGTGGCATCATGTA
IRF8	TGGACATTTCCGAGCCATACA	AGCAGTTGCCACGCCTA
ITGB2	TCAACGAGATCACCGAGTCC	CTTATCAGGGTGCGTGTTCAC
IFNGR1	AAGCCAGGGTTGGACAAAA	GATATCCAGTTTAGGTGGTCCAA
BAX	GGGTTGTCGCCCTTTTCTAC	TCTTGGATCCAGCCCAACA
CASP1	CATTTGAGCAGCCAGATGGTA	GTCCTGGGAAGAGGTAGAAACA
CASP8	GGAAATCTCCAAATGCAAACTGG	CAGGATGACCCTCTTCTCCAT
CCL2	TAGCAGCCACCTTCATTCCC	CCTCTGCACTGAGATCTTCCTA
CCL3	ATGGCTCTCTGCAACCAGTT	CCGGGAGGTGTAGCTGAAG
CXCL1	CTTGCCTCAATCCTGCATCC	AGCCACCAGTGAGCTTCC
BCL2	ATGTGTGTGGAGAGCGTCAA	GTGCCGGTTCAGGTACTCA
GAPDH	GAACGGGAAGCTTGTCATCAA	ATCGCCCCACTTGATTTTGG
TGFB	CGTCTGCTGAGGCTCAAGTTA	TCGCCAGGAATTGTTGCTGTA
S100A12	TTGCTGTAGCTCCACATTCC	TCCCTCCAGATGCTCTTCAA
S100A8	GCTAGAGACCGAGTGTCCTCA	CCAGAATGAGGAACTCCTGGAA
STAT4	CAGTGCTGGAGGTAAAGGAA	AGAGGCAGATCTGTGTTTCAA
STAT5B	AACAGAGGTTGGTCCGAGAA	GTTTCTGGGACATGGCATCA
TNF	CCCAGGGACCTCTCTCTAATCA	ATGGGCTACAGGCTTGTCAC
FASLG	TGGGGATGTTTCAGCTCTTCC	CTGTGTGCATCTGGCTGGTA
FOS	CCCGCAGACTCCTTCTCC	TGGTCGAGATGGCAGTGAC
JUNB	TGGCCCAGCTCAAACAGAA	AGAAGGCGTGTCCCTTGAC
RELB	TGCTTTCCGAGCCCGTCTA	CGGCCCGCTTTCCTTGTTAA
TNFRSF1B	ACATACACCCAGCTCTGGAAC	AGTGCAGGCTTGAGTTTCCA
TNFRSF14	GTAGTCAAGGTGATCGTCTCC	TCAATGACTGTGGCCTCAC
TLR9	TGCAACTGGCTGTTCCTGAA	ACAAGGAAAGGCTGGTGACA
CD163	AGTGCAGAAAACCCCACAA	CAAGGATCCCGACTGCAATA
FCGR1A	AGCAGCTCTACACAGTGGTT	ACTGGCAGAGGTGATTCTGT
FCGR2A	AAGCCTGTGACCATCACTGT	GCAATGACCACAGCCACAAT
FCGR3A	CATTCTTTCCACCTGGGTACCA	AGTCCTGTGTCCACTGCAAA

Supplementary Figure 1. Surface marker expression on monocytes stimulated with hIL10 or vIL10

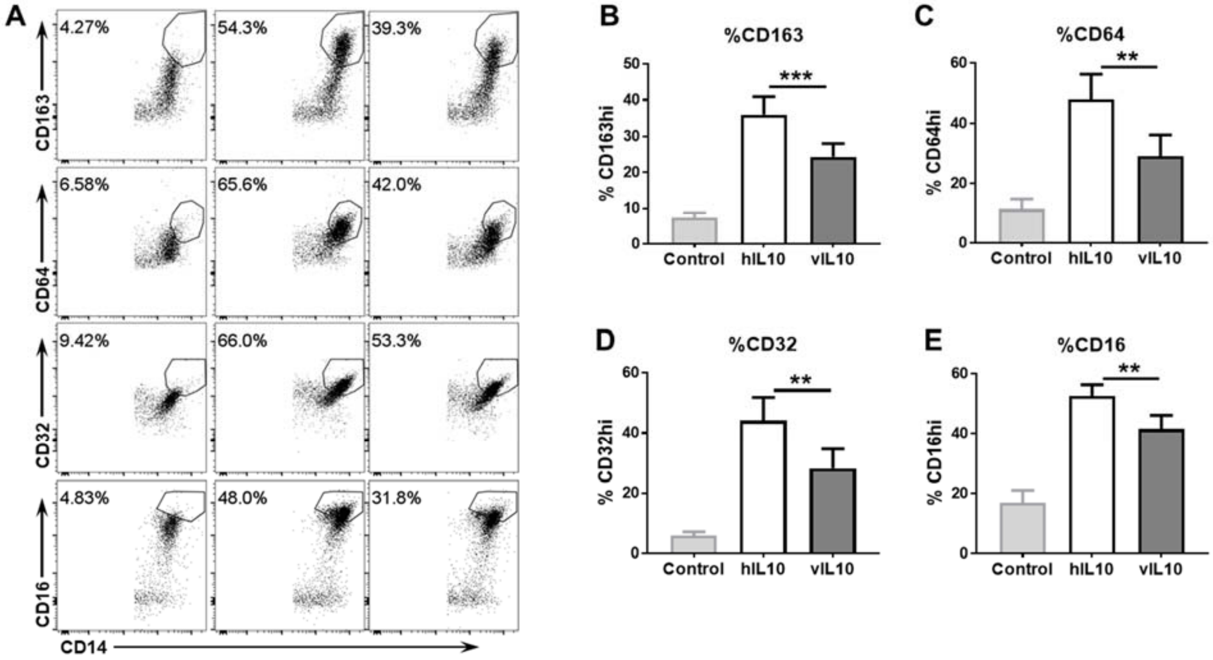

Monocytes were stimulated with 10ng/ml hIL-10 or vIL-10 for 18h, and surface marker expression was determined by flow cytometry. A. Representative dot plots for CD14+ cells expressing different surface markers. Percent of CD14+cells with high expression of **B.** CD163, **C.** CD64, **D.** CD32, and **E.** CD16, are shown. Monocytes stimulated with vIL-10 had smaller increases in CD163, CD64, CD32, and CD16 hi cells when compared to hIL-10**.** *n* =8, ***p*<0.01, ****p*<0.05.

Supplementary Figure 2. vIL-10 levels do not correlate with SLE disease activity

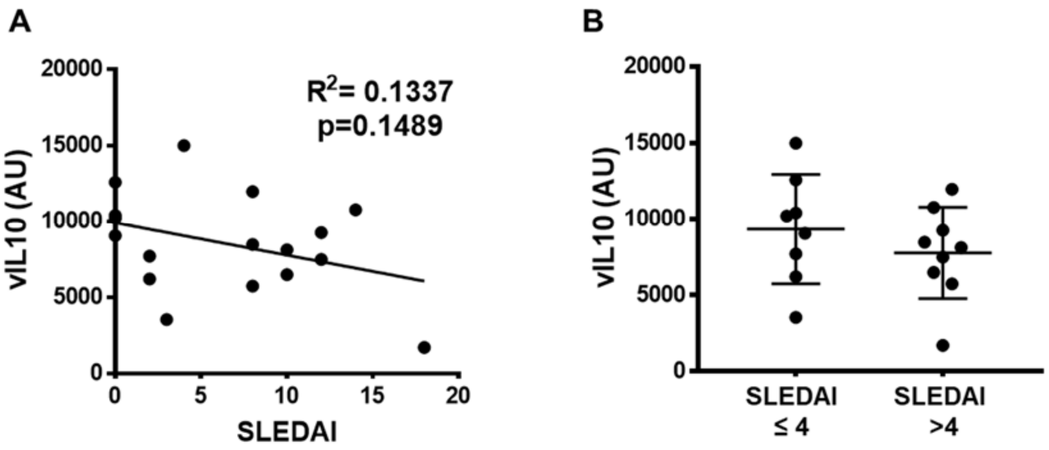

A. Levels of vIL-10 did not show significant correlation with disease activity (SLEDAI). **B.** No significant differences in plasma vIL-10 levels were observed between SLE patients with lower disease activity (SLEDAI ≤ 4) and SLE patients with elevated disease activity (SLEDAI > 4).
